# Treatment of Secondary Aortoenteric Fistulas Following AORTIC Aneurysm Repair in a Tertiary Reference Center

**DOI:** 10.3390/jcm11154427

**Published:** 2022-07-29

**Authors:** Kyriakos Oikonomou, Karin Pfister, Piotr M. Kasprzak, Wilma Schierling, Thomas Betz, Georgios Sachsamanis

**Affiliations:** 1 Department of Vascular and Endovascular Surgery, University Medical Center Regensburg, Franz-Josef-Strauss-Allee 11, 93053 Regensburg, Germany; piotr.kasprzak@ukr.de (P.M.K.); wilma.schierling@ukr.de (W.S.); thomas.betz@ukr.de (T.B.); sachsamanis@hotmail.com (G.S.); 2 Department of Vascular and Endovascular Surgery, Cardiovascular Surgery Clinic, University Hospital Frankfurt and Johann Wolfgang Goethe University Frankfurt, 60596 Frankfurt, Germany

**Keywords:** aortoenteric, aortointestinal, fistula, endovascular aneurysm repair, abdominal aortic aneurysm

## Abstract

Objectives: To present our experience with various therapeutic approaches for the treatment of secondary aortoenteric fistulas following open and endovascular aortic aneurysm repair. Methods and Materials: A retrospective data analysis of patients treated for secondary aortoenteric fistulas in a single vascular institution between January 2005 and December 2018 was performed. Analyzed parameters included patients’ demographics, clinical presentation, diagnostic work-up, perioperative data and repair durability during follow-up. Results: Twenty-three patients with aortoenteric fistulas were treated in the target period. The fistulous connection was located in 21 cases (91.3%) in the duodenum and in two cases (8.7%) in the small intestine. Average time between the initial procedure and detection of the aortoenteric fistula was 69.4 ± 72.5 months. The most common presenting symptom was gastrointestinal bleeding (*n* = 12, 52.2%), followed by symptoms suggestive of chronic infection (*n* = 11, 47.8%). Open surgical repair was performed in 19 patients (bridging in 3 patients), and endovascular repair was carried out in two cases and one patient underwent a hybrid operation. One patient underwent abscess drainage due to significant comorbidities. Mean follow-up was 35.1 ± 35.5 months. In-hospital mortality and overall mortality were 43.5% (10/23) and 65.2% (15/23), respectively. Patients presenting with bleeding had a significantly higher perioperative mortality rate in comparison to patients presenting with chronic infection (66.7% (8/12) and 18.2% (2/11), respectively, *p* = 0.019). Patients who underwent stent-graft implantation for control of acute life-threatening bleeding showed significantly better perioperative survival in comparison to patients that were acutely treated with an open procedure (66.6%, (4/6) and 0% (0/6), respectively, *p* = 0.014). Perioperative mortality was also higher for ASA IV patients (71.4%, 5/7), when compared to ASA III Patients (31.2%, 5/16), although this did not reach statistical significance (*p* = 0.074). Conclusion: Treatment of secondary aortoenteric fistulas is associated with a high perioperative mortality rate. Patients who survive the perioperative period following open surgical repair in the absence of hemorrhagic shock show acceptable midterm results during follow-up. Stent-graft implantation for bleeding control in patients presenting with life-threatening bleeding seems to be associated with lower perioperative mortality rates.

## 1. Introduction

First described in 1818, aortoenteric fistulas (AEFs) represent a rare vascular pathology, referring to the anomalous connection between the aorta and various parts of the gastrointestinal tract [1]. The connection site is most commonly the duodenum, followed by various parts of the ileum [2]. It is a condition associated with considerable mortality rates [2,3]. Secondary AEFs occur mostly after open or endovascular aortic surgery [4]. The incidence of secondary AEFs following open abdominal aortic aneurysm (AAA) and endovascular aneurysm repair (EVAR) ranges between 0.36–1.6% [5]. Chronic graft infection or physical stimulation through aortic pulsation pressure has been suggested to contribute to the formation of secondary AEFs [6]. Clinical presentation can vary, including gastrointestinal bleeding and signs of chronic infection such as weakness, fever or weight loss. In many cases, patients present with acute, life-threatening hemorrhage.

In the last years, various surgical approaches regarding management of secondary AEFs have been reported. This usually involves explantation of the previously implanted material, exclusion of the fistula and open surgical aortic reconstruction or aortic ligation and extra-anatomical bypass. In cases of life-threatening bleeding, deployment of a stent-graft inside the aorta can be considered a bridging operation in order to achieve hemodynamic stabilization of the patient [7,8,9,10]. However, data concerning durability during follow-up and postoperative complications are still inconclusive when it comes to type of reconstruction and material used. The aim of this report is to present our experience with management of secondary AEFs using various therapeutic approaches depending on patients’ medical status and clinical presentation.

## 2. Methods and Materials

### 2.1. Patient Population

We conducted a retrospective data analysis of patients treated for secondary AEF from January 2005 until December 2018. Patients who received surgical or conservative therapy for secondary aortoenteric fistulas were identified and included in the study. Analyzed parameters included patients’ demographics, clinical presentation and medical history. Perioperative outcomes and follow-up data were collected. The institution’s ethical committee has approved this study (registration number 12-101-0121).

### 2.2. Presentation-Diagnosis-Management

Diagnostic work up included clinical and laboratory examinations and imaging identification of the fistula. Radiological examinations prior to operation included computer tomography angiography (CTA) and endoscopy. In semielective cases of possible stent-graft/endograft infection patients, patients underwent an additional PET-CT scan. In some cases of acute hemorrhage, the fistula was identified intraoperatively.

Our protocol for management of a secondary AEF consisted of exclusion of the previously implanted material and restoration of the arterial patency with either aortic reconstruction or an extra-anatomical bypass, while the part of the gastrointestinal tract containing the fistula was either to be sutured or excised. In cases of fistula excision, patients underwent an enteroenteric anastomosis for restoration of the gastrointestinal tract. Omentum wrapping was performed in all cases for additional protection.

In semielective cases of patients presenting with signs of chronic infection, surgical management was planned as a single-stage procedure. In patients who presented with acute gastrointestinal bleeding, stent-graft implantation for damage control was initially performed. This action took place only when the fistula could be identified during preoperative imaging and in cases where straight endograft implantation should suffice for exclusion of the fistula. After achieving hemodynamic stabilization, a standard open repair procedure was planned, with resection of the implanted material—including the stabilization stent-graft, restoration of the arterial patency and management of the fistula.

### 2.3. Follow-Up Data Analysis

Patients were monitored postoperatively with duplex ultrasound (DUS) after six and 12 months and then annually. A CTA scan was carried out after 12 months and then every 2–3 years. In cases of recurrent hemorrhage or signs of infection, the patient was readmitted to the hospital and CTA and PET-CT scans were carried out. Patients that did not attend follow-up sessions were contacted via telephone during data acquisition. Data analysis was performed using SPSS for windows (Version 27.0; SPSS INC, Chicago, IL, USA). In case of normal distribution, variables are presented as mean ± standard deviation (SD) and as median plus range in cases of skewed data distribution. The statistically analyzed data were considered significant for *p* values < 0.05.

## 3. Results

### 3.1. Patient Population

In the study period, a total of 23 patients (20 male, 3 female, mean age 66.1 ± 7 years) were treated in our institution for AEF. Fistulas were located in the duodenum (*n* = 21) and in the small intestine (*n* = 2).

Prior operations included aortobifemoral, aortoiliac bypass or infrarenal tubing (*n* = 11), endovascular repair with EVAR (*n* = 7), open reconstruction for TAAA (*n* = 1) and both open surgical repair and EVAR (*n* = 3). In one case, development of the fistula followed open reconstruction for renal artery aneurysm. The interval between initial operation and fistula formation was 69.4 ± 72.5 months, with no statistical difference between open surgical and endovascular repair (*p* = 0.432). Patient demographics and preoperative risk factors are summarized in Table 1.

### 3.2. Presentation-Diagnosis-Management

**Figure 1:**
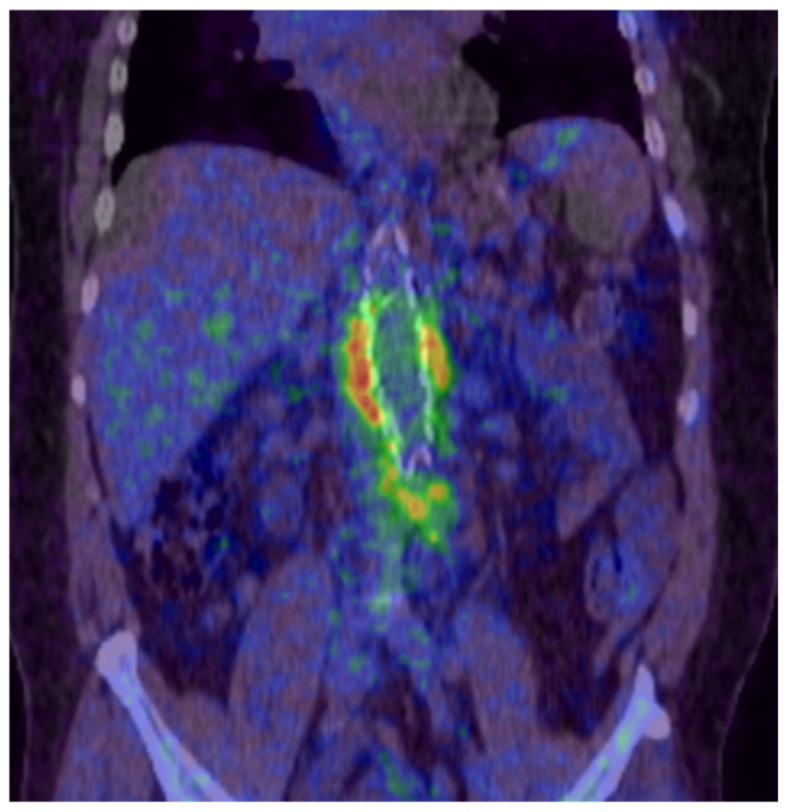
PET-CT scan showing periprosthetic infection of an aortobifemoral bypass (Department of Nuclear Medicine, University Medical Center Regensburg).

**Figure 2:**
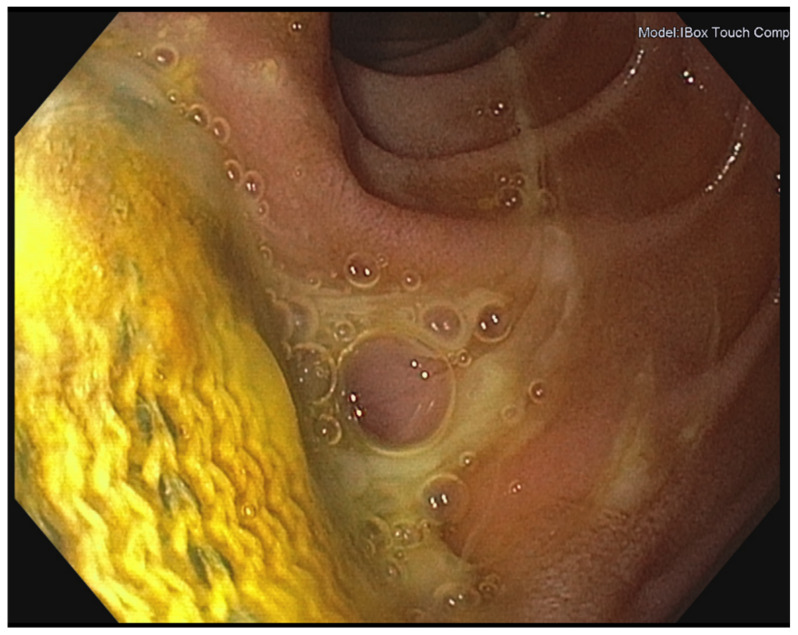
Endoscopic image showing erosion of the prosthetic graft inside the gastrointestinal tract (Department of Internal Medicine I, University Medical Center Regensburg).

Patients presented with bleeding (*n* = 12), followed by signs of chronic infection -fever, weight loss and weakness (*n* = 11). The fistula was identified with CTA in 11 patients (47.8%), endoscopy in 3 (13%), while in 9 patients (39.1%), the fistula was diagnosed intraoperatively (Figure 1 and Figure 2).

Nineteen patients (82.6%) underwent open surgical repair, two patients (8.7%) received endovascular therapy and one patient (4.3%) was treated with a hybrid operation. One patient (4.3%) was deemed unfit for surgery due to multiple comorbidities and underwent a CT-guided abscess drainage.

#### 3.2.1. Open Surgical Repair

Open surgical repair was performed in 19 cases. Sixteen patients underwent primary open surgical repair, and three patients underwent stent-graft implantation as a stabilization procedure prior to open surgery, due to life-threatening hemorrhage.

**Figure 3:**
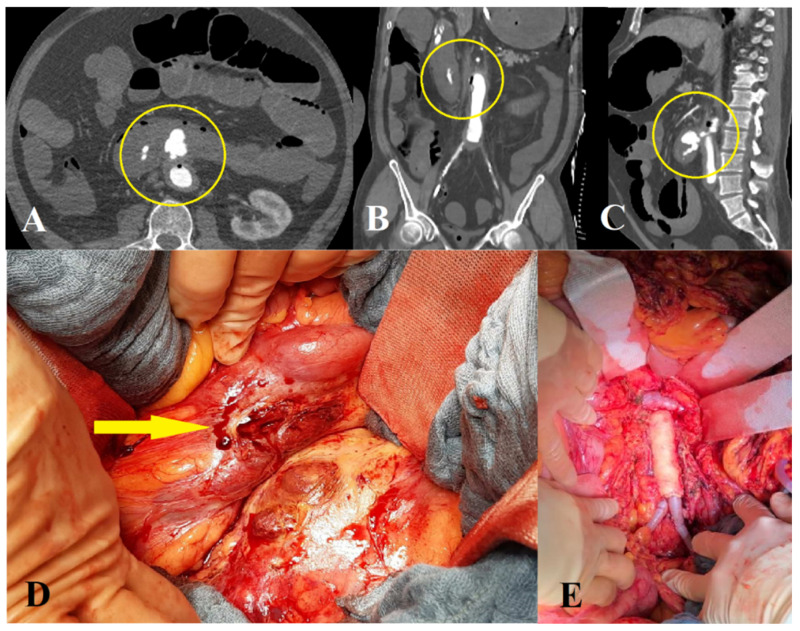
Open surgical repair of a patient with a secondary aortoenteric fistula. Axial (**A**), coronary (**B**) and sagittal (**C**) views of a computer tomography angiography showing contrast agent in the duodenum and air inside the aorta (Department of Radiology, University Medical Center Regensburg). (**D**) Arrow showing fistulous connection point. (**E**) Aortic reconstruction with homograft interponation.

Twelve patients underwent aortic reconstruction and two were treated with an extra-anatomical bypass. In three cases, treatment consisted only of excision and suture of the fistula. Two patients deceased intraoperatively following laparotomy due to massive bleeding, prior to aortic reconstruction. Either autologous vein grafts, prosthetic grafts or homografts were used (Figure 3).

Complete resection of the previously implanted material was performed in 9 of 19 cases, including the 3 patients who received a stent-graft implantation for bleeding control. In five patients, the previous prosthetic material was partly removed. In three patients, no explantation was performed. Two of these patients underwent only fistula management due to significant comorbidities. The third patient presented with hemorrhagic shock due to erosion bleeding two months after open repair of an AAA. Management consisted of fistula suture and restoration of the proximal anastomosis with a salvaging of the previous implanted aortobifemoral prosthesis.

Ten patients underwent primary and seven patients secondary closure of the abdominal cavity. Reoperation time was one to three days. In cases of primary abdominal closure, a continuous suture technique was used. Two patients with secondary abdominal closure had additional mesh reinforcement. A summary of open surgical repair can be found in Table 2.

#### 3.2.2. Endovascular Repair

**Figure 4:**
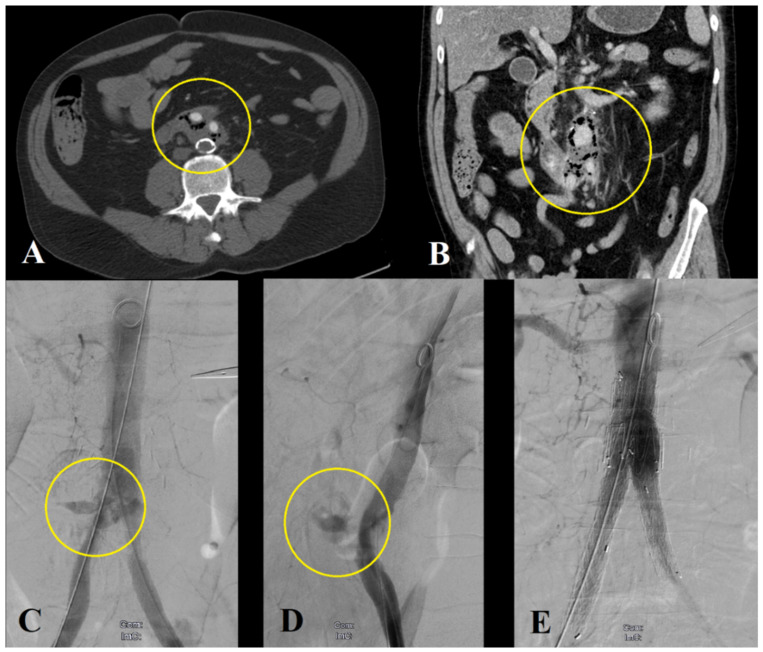
Emergency endovascular management of a patient with acute life-threatening bleeding due to an aortoenteric fistula. Computer tomography angiography showing air inside the aortic wall (**A**,**B**), (Department of Radiology, University Medical Center Regensburg). Stabilization of the patient with stent-graft implantation in the aorta and the iliac arteries (**C**–**E**).

Two patients underwent endovascular management of AEF with infrarenal stent-graft implantation without subsequent open repair. Both patients presented with acute gastrointestinal bleeding, were unfit for open surgery due to significant comorbidities or malignancy and were treated endovascularly to contain the acute bleeding (Figure 4).

#### 3.2.3. Hybrid Repair

One patient with aortoduodenal fistula following fenestrated EVAR underwent a hybrid operation. This involved a relining of the endovascular graft and overstenting of all target vessels in order to contain the acute bleeding. Further management included visceral debranching and removal of the fistulous part of the bowel via laparotomy. The patient had a primary abdominal closure using a continuous suture technique.

### 3.3. Perioperative Mortality and Morbidity

Mean ICU and hospital stay were 9.2 ± 8.8 and 22.3 ± 17.8 days, respectively. Overall, in-hospital mortality was 43.5% (10/23).

Regarding operative management, the mortality of patients who underwent open surgical repair was 42.1% (8/19). Patients who received endovascular management showed a perioperative mortality rate of 50% (1/2). The patient who had an abscess drainage became deceased 14 days after hospital admission. There was no statistical significance between patients’ mortality according to type of material used during open repair (*p* = 0.128). Additionally, there was no statistical significance regarding the type of reconstruction (*p* = 0.241). Patients who underwent complete resection of the previously implanted material showed lower mortality rates compared to patients who underwent only partial or no material resection, although this did not reach statistical significance (2/9, 22.2% versus 4/8 50%, respectively, *p* = 0.201). Patients’ postoperative causes of death according to operative management are summarized in Table 3.

Patients presenting with bleeding had a statistically higher perioperative mortality rate in comparison to patients presenting with signs of chronic infection (66.7% (8/12) and 18.2% (2/11), respectively, *p* = 0.019). Additionally, patients presenting with acute life-threatening bleeding showed a significantly better perioperative survival when undergoing primary stent-graft implantation for control of the hemorrhage (staged endovascular to open repair, *n* = 3; endovascular repair, *n* = 2; hybrid repair, *n* = 1) in comparison to patients who received immediate open surgical repair (66.6%, (4/6) and 0% (0/6), respectively, *p* = 0.014).

Perioperative mortality was higher for ASA IV patients (71.4%, 5/7) when compared to ASA III Patients (31.2%, 5/16), although this did not reach statistical significance (*p =* 0.074). Complications during hospitalization requiring reintervention included lower extremity ischemia (*n* = 3), insufficiency of gastric anastomosis (*n* = 2), insufficiency of the sutured fistula (*n* = 2), abdominal compartment (*n* = 2) and hemothorax (*n* = 1). In the two cases of gastric anastomosis insufficiency, patients had a new gastric anastomosis, while the two patients with insufficiency of the fistula suture underwent complete resection of the bowel part containing the fistula and had an enteroenteric anastomosis.

### 3.4. Follow-Up

Average follow-up duration was 35.1 ± 35.5 months. Five patients died during follow-up (38.5%, 5/13).

Three patients died during the first, second and third postoperative month because of recurrent bleeding. The other two patients died of nonfistula related causes during follow-up. For the remaining patients, no fistula-related reinterventions were recorded during follow-up. Mortality during follow-up is summarized in Table 4.

## 4. Discussion

Secondary AEFs are a rare vascular condition. Due to the significant comorbidities, these patients usually exhibit an acute presentation, and AEFs are associated with considerable perioperative mortality up to 50% [6,11]. Despite a number of proposed therapeutic approaches, a clear therapeutic plan is yet to be defined. There are various techniques described in the literature, including in situ reconstruction with prosthetic materials or deep femoral vein reconstruction with the neo-aorto-iliac system (NAIS), while other authors propose graft excision and extra-anatomic bypass [7,12,13]. The significantly worse outcome of patients presenting in hemorrhagic shock and the development of endovascular techniques in the last several years raises the question of whether stent-graft implantation can effectively minimize the perioperative risk in patients presenting with acute life-threatening bleeding [14].

In a recent review by Kakkos et al., endovascular repair of AEFs demonstrated better in-hospital mortality rates when compared to open surgery (7.1% to 33.9%, respectively) [2]. Despite that promising early result, 2-year survival rate was 51% and 40%, respectively, mainly due to recurrent bleeding or sepsis. It should be noted that there was a certain bias toward early mortality reported in case reports (16.4% for case reports, 41.5% for case-controlled studies and 31% for case series, *p* < 0.001), in which an endovascular approach was also mostly reported (26.4% in case reports, 8.4% for case series and 8.5% for case control studies, *p* < 0.001) [2]. Another review by Antoniou et al. showed an early mortality of 7% (3/41) in patients who had endovascular repair of an AIF. Of these patients, 44% developed a persistent, recurrent or new infection after a mean follow-up period of 13 months [3]. In our series, two patients received only endovascular therapy (single-staged endograft implantation). One patient died perioperatively and a second patient died during the second postoperative month due to recurrent bleeding. In our experience, endovascular single-stage management cannot be considered to be a long-term solution for aortoenteric fistulas. Patients who received single-stage open surgical repair demonstrated acceptable midterm results in the absence of preoperative hemodynamic instability with a perioperative mortality of 10% (1/10) and late mortality of 33.3% (3/9). Stent-graft implantation may offer a viable first-step option in patients presenting with acute life-threatening bleeding, allowing for hemodynamic stabilization and a definitive open surgical repair at a second stage. In our study, 6 patients out of 12 who presented with acute bleeding underwent stent-graft implantation for control of the bleeding. Postoperative mortality in this subgroup was 33.3% (2/6). Patients presenting with bleeding shock demonstrated a significantly worse perioperative outcome when undergoing immediate open repair (100% mortality, 6/6). Our results are in accordance with the review from Kakkos et al. in which bridging the endovascular approach to open repair demonstrated an in-hospital mortality of 0% (0/13) [2].

The limitations of this study include the small number of patients in conjunction with the different types of operative approaches. Furthermore, there may be a negative outcome bias since the complexity of this vascular condition along with the patients’ significant comorbidities sometimes lead to delayed admission to tertiary reference centers.

## 5. Conclusions

Treatment of aortoenteric fistulas is associated with a high perioperative mortality rate. Patients who survive the perioperative period following open surgical repair in the absence of hemodynamic instability show acceptable midterm results during follow-up. Stent-graft implantation for bleeding control prior to explantation in patients presenting with life-threatening bleeding seems to be associated with lower perioperative mortality rates. More data and larger patient cohorts are needed in order to draw safer conclusions.

## Figures and Tables

**Table 1:** Patient demographics. No = number; y.o = years old; SD = standard deviation; EVAR = endovascular aneurysm repair; TAAA = thoracoabdominal aortic aneurysm; ASA; American Society of Anesthesiology score; CAD = coronary artery disease; COPD = chronic obstructive pulmonary disease; CKD = chronic kidney disease.

Variable	No. (Percent)
**Male/Female ratio**	20 (87%)/3 (13%)
**Mean patient age (y.o ± SD)**	66.1 ± 7
**Fistula localization**	
Duodenum	21 (91.3%)
Small intestine	2 (8.7%)
**Prior operation type**	
Aortobifemoral bypass	5 (21.7%)
Aortobiiliac bypass	3 (13%)
Infrarenal tubing	3 (13%)
EVAR	7 (30.4%)
Open repair + EVAR for aneurysmal management	3 (13%)
Open repair for TAAA	1 (4.3%)
Open repair for renal artery aneurysm	1 (4.3%)
**ASA Grading**	
ASA III	16 (69.6%)
ASA IV	7 (30.4%)
**Commorbidities**	
CAD	12 (52.2%)
Hypertension	14 (60.9%)
COPD	5 (21.7%)
Diabetes	6 (26.1%)
Smoking	8 (34.8%)
Hypercholesterolemia	8 (34.8%)
CKD	11 (47.8%)

**Table 2:** Open surgical repair of patients with secondary aortoenteric fistulas.

Type of Repair	No. (Percent)
Aortic interponation	10 (71.4%)
Aortobifemoral bypass	1 (7.1%)
Aortobiiliac bypass	1 (7.1%)
Axillobifemoral bypass	2 (14.3%)
**Type of material used**	
Autologous	2 (14.3%)
Prosthetic	10 (71.4%)
Homograft	2 (14.3%)
**Prosthetic material**	
Removed	9 (53%)
Partially removed	5 (29.4%)
Left in situ	3 (17.6%)
**Fistula**	
Excised	4 (23.5%)
Sutured	13 (76.5%)

**Table 3:** Postoperative causes of death according to type of treatment.

Type of Treatment	Patients
**Open surgical repair**	
Multiorgan failure	3
Colonic ischemia	2
Cardiac failure	1
**Endovascular repair**	
Aortic rupture	1
**Abscess Drainage**	
Multiorgan failure	1

**Table 4:** Mortality of patients who survived the perioperative period, depending on type of repair and fistula relation.

Type of Repair	Follow-Up Mortality	
	Fistula Related	Non-Istula Related
**Open Repair (with Bridging)**	1/3 (33%)	0/3 (0%)
**Open Repair (without Bridging)**	1/9 (11%)	2/9 (22%)
**Endovascular**	1/1 (100%)	0/1 (0%)

## Data Availability

The data presented in this study are available on request from the corresponding author. The data are not publicly available due to ethical and privacy compliance.
